# Differentiating Encephalopathy From Seizure in Organophosphate Poisoning: Utility of a Benzodiazepine Trial and Risk Scoring

**DOI:** 10.7759/cureus.99323

**Published:** 2025-12-15

**Authors:** Tetsuya Oyama, Hirotaka Nakanishi

**Affiliations:** 1 Neurology, Yokkaichi Municipal Hospital, Mie, JPN

**Keywords:** 2helps2b score, benzodiazepine trial, diagnostic dilemma, generalized periodic discharges, nonconvulsive status epilepticus, organophosphate pesticide poisoning, triphasic waves

## Abstract

Organophosphate poisoning can present with generalized periodic discharges (GPDs) on electroencephalography (EEG), posing a diagnostic challenge in differentiating toxic metabolic encephalopathy (TME) from nonconvulsive status epilepticus (NCSE). We report the case of a 76-year-old woman with organophosphate poisoning who presented with coma and 2.0 Hz triphasic GPDs. A diagnostic benzodiazepine trial resulted in electrographic resolution without clinical improvement, and the 2HELPS2B risk score was 0. These findings supported a diagnosis of nonictal TME, allowing for the avoidance of aggressive antiseizure medication. This case highlights the utility of a structured approach using a benzodiazepine trial and risk stratification scoring to guide management in ambiguous EEG patterns associated with organophosphate poisoning.

## Introduction

Continuous electroencephalography (CEEG) is essential for detecting nonconvulsive status epilepticus (NCSE) [1]. However, it occasionally shows generalized periodic discharges (GPDs), particularly in toxic metabolic encephalopathy (TME), which present two clinical challenges. The first is diagnostic: GPDs in TME can mimic or coexist with NCSE, making accurate differentiation critical [2]. The second is therapeutic: the management of nonictal patterns is unestablished [2]. In clinical practice, typical strategies to address this dilemma include careful correlation with clinical signs, assessment of EEG features such as evolution or plus modifiers, diagnostic benzodiazepine trials, and the use of seizure-risk stratification tools. We report a case in which a negative benzodiazepine trial confirmed a non-epileptic etiology, and the 2HELPS2B score guided subsequent management.

## Case presentation

**Figure 1:**
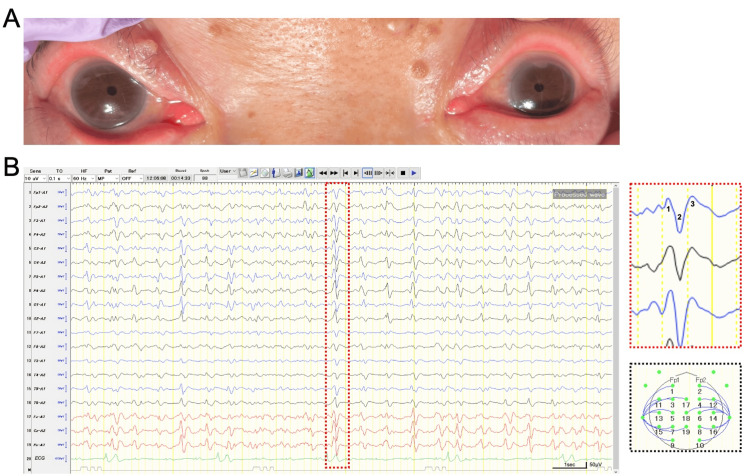
Clinical signs and electroencephalographic findings. (A) Bilateral miosis (pinpoint pupils) observed on admission, a classic physical sign supporting the diagnosis of organophosphate poisoning. (B) EEG showing 2.0Hz GPDs with classic triphasic morphology. The right panels provide detailed views: the upper right panel highlights a single triphasic wave, with numbers one, two, and three indicating the three characteristic phases, and the lower right panel displays the referential montage used in this recording. EEG, electroencephalogram; GPDs, generalized periodic discharges

A 76-year-old woman with bipolar disorder was admitted following pesticide ingestion in a suicide attempt. The patient was comatose with a Glasgow Coma Scale score of 6 (E1V1M4), pinpoint pupils (<1 mm) (Figure 1A), a characteristic odor, and excessive secretions. A head computed tomography scan performed on admission revealed no acute structural abnormalities. A low serum cholinesterase level of 4 U/L (reference range: 201-421 U/L) supported the clinical diagnosis of organophosphate poisoning. The patient was intubated and received continuous infusions of atropine and pralidoxime. Atropine was initiated at 0.25 mg/kg/h (body weight 40 kg) and titrated based on the resolution of parasympathetic symptoms, such as airway secretions. Pralidoxime was administered at 200 mg/h for 48 hours.

Despite hemodynamic stabilization, the patient’s profound coma persisted. Brain magnetic resonance imaging was not performed due to the patient’s unstable respiratory condition. Consequently, on hospital day 8, an EEG was recorded to investigate the cause of the coma. Following the recording of 2.0 Hz GPDs with triphasic morphology (Figure 1B), a diagnostic trial with intravenous diazepam (5 mg) was immediately performed. This resulted in electrographic resolution of the GPDs; however, no corresponding clinical improvement or change in the level of consciousness was observed. The pattern was therefore deemed nonictal, and supportive care was continued. Around hospital day 11, the patient’s consciousness began to improve, allowing for extubation. However, the patient subsequently developed respiratory failure, and after the family declined reintubation, she died on day 15.

## Discussion

From this case, we learned several important lessons. First, GPDs in TME can mimic NCSE. Second, a benzodiazepine trial is a valuable tool for this specific diagnostic problem. Finally, the management of GPDs with triphasic morphology, once presumed to be nonictal, requires a case-by-case approach.

According to the 2021 American Clinical Neurophysiology Society (ACNS) terminology, GPDs, which reflect widespread cerebral dysfunction, are associated with various conditions, including TME [3]. This creates a diagnostic dilemma because GPDs can also represent NCSE, and TME can complicate NCSE. While features such as frequency, evolution, or plus modifiers aid differentiation, waveform analysis alone is sometimes insufficient [4]. Our patient's EEG showed 2.0Hz GPDs without evolution or plus modifiers, classifying the pattern on the ictal-interictal continuum (IIC) [3].

When the EEG is ambiguous, correlation with clinical findings is critical to differentiate TME from NCSE. The 2021 ACNS criteria define an electroclinical seizure (ECSz) as a pattern time-locked to a clinical event or with both EEG and clinical improvement after an intravenous antiseizure medication [3]. Our patient lacked a clear time-locked clinical correlate. A diagnostic benzodiazepine trial was performed to differentiate the etiology. This procedure involves the administration of a benzodiazepine, in this case, a 5 mg diazepam bolus, under continuous EEG monitoring to assess for the resolution of rhythmic or periodic patterns and any concurrent clinical improvement. In our case, the trial resulted in electrographic improvement without clinical change. As this response did not meet the criteria for definitive ECSz, we considered the GPDs to be nonictal [5]. However, the absence of clinical improvement should be viewed with caution, as profound toxic-metabolic coma may obscure any immediate response to seizure suppression.

Given this diagnostic uncertainty, even when GPDs are presumed to be nonictal, their management is complicated by their potential to evolve into seizures. This is particularly controversial for GPDs with triphasic morphology, as seen in our patient [6,7]. While this pattern was traditionally associated with TME and considered to have a low seizure risk, one study found the seizure risk to be comparable to that of other GPDs [6]. Lacking definitive guidelines, a case-by-case decision is necessary, guided by risk stratification scores such as the 2HELPS2B score [8]. The 2HELPS2B score consists of six EEG and clinical features, including prior seizure history, epileptiform discharges, lateralized periodic discharges, plus features, frequency >2Hz, and brief potentially ictal rhythmic discharges (weighted 2 points). Scores range from 0 to 7 and correlate with seizure probability. A score of 0 is associated with a seizure risk of <5%, and prolonged antiseizure treatment is generally not recommended [8]. In our patient, a score of 0 indicated a very low seizure risk, providing objective support for withholding antiseizure treatment. This assessment allowed us to avoid unnecessary therapeutic escalation.

## Conclusions

In conclusion, this case illustrates that when GPDs raise diagnostic uncertainty between NCSE and TME, a structured multimodal approach is essential. In our patient, a diagnostic benzodiazepine trial demonstrated electrographic improvement without clinical change, favoring a nonictal etiology. Furthermore, a 2HELPS2B score of 0 indicated a very low seizure risk (<5%), providing additional support for withholding antiseizure treatment. These complementary assessments allowed us to appropriately classify the GPDs as nonictal and avoid unnecessary therapeutic escalation.
